# Identification of a Conserved Linear B-Cell Epitope of *Streptococcus dysgalactiae* GapC Protein by Screening Phage-Displayed Random Peptide Library

**DOI:** 10.1371/journal.pone.0131221

**Published:** 2015-06-29

**Authors:** Limeng Zhang, Hua Zhang, Ziyao Fan, Xue Zhou, Liquan Yu, Hunan Sun, Zhijun Wu, Yongzhong Yu, Baifen Song, Jinzhu Ma, Chunyu Tong, Xintong Wang, Zhanbo Zhu, Yudong Cui

**Affiliations:** 1 College of Life Science and Technology, HeiLongJiang BaYi Agricultural University, Daqing, P. R. China; 2 College of Animal Science and Veterinary Medicine, HeiLongJiang BaYi Agricultural University, Daqing, P. R. China; National Cancer Institute, NIH, UNITED STATES

## Abstract

The GapC of *Streptococcus dysgalactiae* (*S*. *dysgalactiae*) is a highly conserved surface protein that can induce protective humoral immune response in animals. However, B-cell epitopes on the *S*. *dysgalactiae* GapC have not been well identified. In this study, a monoclonal antibody (mAb5B7) against the GapC_1-150_ protein was prepared. After passive transfer, mAb5B7 could partially protect mice against *S*. *dysgalactiae* infection. Eleven positive phage clones recognized by mAb5B7 were identified by screening phage-displayed random 12-peptide library, most of which matched the consensus motif DTTQGRFD. The motif sequence exactly matches amino acids 48-55 of the *S*. *dysgalactiae* GapC protein. In addition, the motif ^48^DTTQGRFD^55^ shows high homology among various *streptococcus* species. Site-directed mutagenic analysis further confirmed that residues D48, T50, Q51, G52 and F54 formed the core motif of ^48^DTTQGRFD^55^. This motif was the minimal determinant of the B-cell epitope recognized by the mAb5B7. As expected, epitope-peptide evoked protective immune response against *S*. *dysgalactiae* infection in immunized mice. Taken together, this identified conserved B-cell epitope within *S*. *dysgalactiae* GapC could provide very valuable insights for vaccine design against *S*. *dysgalactiae* infection.

## Introduction

As one of the most important pathogens causing mastitis, the *Streptococcus dysgalactiae* (*S*. *dysgalactiae*) is intimately associated with intramammary infection in the dairy cow [1]. This pathogen is ubiquitous in the dairy environment and can survive for long periods only within the mammary gland [2]. The *S*. *dysgalactiae* expresses various intracellular, extracellular or cell surface proteins, which specifically interacts with host proteins. These interactions are assumed to play important roles in eliciting host immune reactivity [3–5].

Nowadays, whole organism [6], capsular carbohydrate [7], or recombinant proteins [5, 8–11] have been developed as potential vaccines. Especially, several surface proteins have been used as recombinant vaccine components, and their partial protection effects against the *streptococcus* infection have been achieved [3, 8, 12]. One of these surface proteins is the GapC protein, which was first identified in Group A streptococci (GAS). It is the streptococcal surface dehydrogenase (SDH) [5, 13]. SHS possesses activity of the Glyceraldehyde 3-phosphate dehydrogenase (GAPDH). This key enzyme in the glycolysis cycle of prokaryotic and eukaryotic cells reversibly catalyzes the conversion of glyceraldehyde 3-phosphate to 1, 3 bi-phosphoglycerate [14–16]. GAPDH is also a stimulatory protein that induces the proliferation and differentiation of B cells by inducing IL-10 production [17]. The GapC in different *S*. *dysgalactiae* species shares considerable homology at the DNA and amino acid levels [10], suggesting that GapC protein might be a good immunodominant antigen. The GapC protein functions as an immunodominant protein and is responsible for eliciting antibodies against *S*. *dysgalactiae* [18]. It is well known that antigen elicits immune responses mainly through its epitopes, such as B-cell epitopes. B-cell epitopes are defined as regions on the surface of the native antigen that are recognized by binding to B-cell receptors or specific antibodies [19]. Up to now, the B-cell epitopes on *S*. *dysgalactiae* GapC protein and its core sequence have not been well characterized.

Our previous study suggested that the fragment of 1 to 150 amino acids located at the N-terminus of *S*. *dysgalactiae* GapC protein could induce same immune response as the full-length GapC protein [18]. Thus in this study, the truncated GapC protein, which we named GapC_1-150_, was used as the immunodominant fragment. For the sake of increasing solubility of recombinant protein, the GapC_1-150_ was firstly expressed as a His-TrxA fusion protein. And this fusion protein was successfully purified by Ni-NTA purification system [18]. Then the neutralizing monoclonal antibody 5B7 (mAb5B7) against GapC_1-150_ protein of the *S*. *dysgalactiae* was generated and characterized. The precise B-cell epitope ^48^DTTQGRFD^55^ located in the N-terminus of the GapC protein was mapped through screening a phage-displayed random 12-mer peptide library. Its core motif D^48^T^50^Q^51^G^52^F^54^ was further identified using site-directed mutagenic analysis. These findings will aid in the further study of GapC epitope-vaccines against *S*. *dysgalactiae*.

## Materials and Methods

### Animals, ethics statement and production of anti-GapC mouse serum

BALB/c mice (SPF, 4–6 weeks, 18–22 g) were provided by Experimental Animal Center, Harbin Veterinary Research Institute of the Chinese Academy of Agricultural Sciences. The criteria used to determine when the animals should be humanely sacrificed includes weight loss, inappetence, weakness or inability to obtain feed or water, signs of severe organ system dysfunction, non-responsive to treatment, serve infection. Care of laboratory animals and animal experimentation were performed in accordance with animal ethics guidelines and approved by the Animal Ethics Committee of HeiLongJiang BaYi Agricultural University. The anti-GapC mouse serum was prepared in our lab, and was identified using indirect enzyme-linked immunosorbent assay (ELISA). BALB/c mice were immunized subcutaneously with 50 μg of GapC emulsified with ISA50 V2 (SEPPIC, France), followed by three boosts with the same dose at 2-week intervals. Twenty one days after the final boost, the BALB/c mice were anesthetized, humanely sacrificed and blood. The anti-GapC mouse serum was isolated from coagulated blood, and the concentration of anti-GapC immunoglobulins in this serum was determined about 100 μg/ml according to our previous study [8]. The 96-well microtitre plates were coated overnight at 4°C with *S*. *dysgalactiae* GapC, and blocked with 200 μl of PBST for 1 h at 37°C. A total of 100 μl of anti-GapC mouse serum was added, and plates were incubated for 2 h at 20°C. After washing, HRP-conjugated goat anti-mouse IgG was added, and plates were incubated for 1 h at room temperature. Plates were washed, and optical density (OD) value of each well was detected at 450 nm at room temperature.

### Plasmid, cell lines and bacterial strains

To construct full-length and truncated (1-150aa) GapC of *S*. *dysgalactiae*, DNA fragment was cloned into pET-32a(+) plasmid resulting in the corresponding His-TrxA fusion protein. All of *Streptococcus uberis* (*S*. *uberis*), *Streptococcus agalactiae* (*S*. *agalactiae*) and *Staphylococcus aureus* (*S*. *aureus*) *gapC* genes were cloned into pET-30a(+) plasmid resulting in the His fusion proteins, respectively. The myeloma cell line SP2/0 was maintained in Dulbecco’s Modified Eagle’s Medium supplemented with 10% fetal calf serum (HyClone, USA) and 1% penicillin-streptomycin. Strains of *S*. *dysgalactiae* LS0312 (GenBank accession number: 30348860), *S*. *agalactiae* LS0310 (GenBank accession number: 21666598), *S*. *uberis* SD0306 (GenBank accession number: 2166660) were stored in our laboratory.

### Expression and purification of recombinant proteins

Recombinant protein was expressed in *E*. *coli* strain BL21 (DE3). After the competent cells harboring the recombinant plasmid were cultivated to an A_600_ of 0.6 to 0.8 in LB medium at 37°C, 0.8 mM isopropyl-β-D-1-thiogalactopyranoside (IPTG) was added to the medium to induce recombinant protein expression for 3 h. Then the cells were harvested and resuspended in phosphate-buffered saline (PBS, pH7.4). The cells were disrupted by ultrasonication, and the supernatant containing soluble recombinant protein was collected. The protein with 6 × His tag was purified with Ni-NTA purification system (Merck, Germany) according to the manufacturer’s instruction. The expressed recombinant protein and its purity were analyzed by SDS-PAGE and Western blot.

### Preparation and purification of mAb

Monoclonal antibodies (mAbs) against the recombinant GapC_1-150_ were produced using a standard procedure [20, 21]. Briefly, 4–6 weeks old BALB/c female mice were immunized by subcutaneous injection with 100 μg of the purified recombinant GapC_1-150_ emulsified with an equal volume of Freund’s complete adjuvant, then followed by two injections at 2-week intervals with the protein emulsified incomplete adjuvant [22]. Three days later, the mice were intraperitoneally injected with 50 μg of the antigen alone. The immunized BALB/c mice were sacrificed by cervical dislocation, and the splenic cells were harvested and fused with the SP2/0 mouse myeloma cells. The fused cells were cultured and selected in 1640 medium (HAT medium and HT medium) [23]. The mAb-producing hybridoma was cloned by limiting dilution of the cells three times. Mice were primed with a 0.5 ml adjuvant and then injected with 1–3 × 10^6^ hybridoma cells by intraperitoneal injection. The mAbs were purified from the mice ascetic fluid using caprylic acid/ammonium sulfate precipitation (CA-AS) and the Nab Protein G Spin Purification Kit (Thermo, USA).

### Characterization of mAb5B7

The mAb5B7 titer of culture supernatant or the ascites fluid was determined by indirect ELISA. Briefly, a 96-well plate was coated with 5 μg/ml of purified recombinant GapC_1-150_ at 4°C overnight and blocked with 5% skimmed milk dissolved in PBS at 37°C for 1 h to prevent non-specific protein binding. Then, 100 μl of supernatant of mAb-producing hybridoma culture or the ascetic fluid was added to wells and incubated at 37°C for 1 h. After washing three times with PBST, HRP-conjugated goat anti-mouse IgG (Sigma-Aldrich) or SBA Clonotyping System/HRP (Southern Biotechnology Associates Inc., USA) was incubated for 1 h at 37°C. Estimation of the enzymatic activity was carried out with TMB as the substrate. The reaction was stopped with 2 M H_2_SO_4_ and the optical density (OD) value of each well was read at 450 nm using a microplate reader (BioRad 550).

The isotype of mAb5B7 was determined using a Mouse Monoclonal Antibody Isotyping Kit (Promega, USA). The mAb specificity for binding recombinant GapC_1-150_ was detected by Western-blot using mAb5B7 as the primary antibody as described above. Reactivity of mAb5B7 with the whole cells of *S*. *dysgalactiae*, *S*. *agalactiae* and *S*. *uberis* were respectively determined by indirect ELISA with a 96 well-plate coated with inactivated whole bacteria.

### Passive immunization with mAb5B7 in mouse

Passive immunization of mAb5B7 against *S*. *dysgalactiae* infection was performed in the following method: four-weeks-old female BALB/c mice were passively immunized intravenously with 200 μg of purified mAb5B7. Control mice were immunized with same volume of SP2/0, respectively (n = 10). Twenty-four hours later, the mice were intraperitoneally challenged with 5 × 10^7^ CFU/mouse of *S*. *dysgalactiae*. Survival was monitored at daily intervals for 7 days after the challenge.

### Screening a random phage-displayed peptide library

The Ph.D.-12 Phage Display Peptide Library (NEB, Beverly, MA, USA) containing random combination of 12-mer peptides was screened with mAb5B7 according to the manufacturer’s instruction and previous study [22]. The 96-well plate was coated with 10 μg/ml mAb5B7 (100 μl/well) at 4°C for 12 h. The well was then washed with TBST for 10 times, filled with 400 μl of blocking buffer and incubated at 4°C for 1 h. The 10 μl phage (1.5 × 10^11^) in 100 μl TBST was incubated and slightly shaking with the well-bound mAb at 20°C for 1 h. After TBST washing, the bound phages were then eluted with 100 μl elution buffer from the well. After neutralization with 15 μl of Tris-HCl, 1 μl phage solution was treated for dilution with LB medium and the titer of the phages was determined. Then the remaining elute was amplified by infecting 20 ml of 1:100 dilution of culture of *E*. *coli* ER2738. The culture was incubated with vigorous vibrating at 37°C for 4 h. At last, the supernatant was colleted and precipitated with PEG/NaCl. The phage yield rate was counted by the following calculation: recovery = (eluted phage/input phage) × 100%. After three rounds of repeat biopanning, positive phage clones were randomly selected and their reactivity to the mAb5B7 was verified by sandwich ELISA. The single strand phage DNAs of the positive phage clones verified by sandwich ELISA were extracted and sequenced with primer 5’-TGAGCGGATAACAATTTCAC-3’ as described by the manufacturer’s instructions (New England Biolabs, UK). The phage peptide sequences were deduced from the DNA sequences and aligned with the GapC sequence using the MEGALIGN programme in DNAStar software.

### Precise defining of the epitope with mutagenic analysis

To determine the crucial amino acids of the epitope, each amino acid residue of the epitope was substituted with alanine. A series of complementary oligonucleotides coding for the wild-type and mutated versions of the epitope motif were synthesized by GENEWIZ and were cloned into pGEX-6p-1 vector (Table 1), resulting in a series of recombinant plasmids. The recombinant plasmids were induced to express recombinant GST-fusion proteins. The mAb5B7 was used as primary antibody, and reactivities of various GST fusions with mAb5B7 were detected using Western blot. At the same time, we have mutated each residue of the epitope to alanine in the full-length GapC, and the binding affinities with mAb5B7 were determined in accordance with the mutation of epitope peptide.

**Table 1:** The oligonucleotides coding the truncated GapC_1-150_ N-terminus and alanine-scanning peptides.

Coding motifs	Name	The sequences of oligonucleotides
^46^KYDTTQGRFD^55^	WT-S	5’-gatccaaatatgacacaactcaaggtcgtttcgactaac-3’
	WT-R	5’-tcgagttaGTCGAAACGACCTTGAGTTGTGTCATATTTg-3’
^46^ **A**YDttqgrfd^55^	K46A-S	5’-gatccGCGtatgacacaactcaaggtcgtttcgactaac-3’
	K46A-R	5’-tcgagttaGTCGAAACGACCTTGAGTTGTGTCATACGCg-3’
^46^K**A**DTtqgrfd^55^	Y47A-S	5’-gatccaaaGCGgacacaactcaaggtcgtttcgactaac-3’
	Y47A-R	5’-tcgagttaGTCGAAACGACCTTGAGTTGTGTCCGCTTTg-3’
^46^KY**A**tTqgrfd^55^	D48A-S	5’-gatccaaatatgCGacaactcaaggtcgtttcgactaac-3’
	D48A-R	5’-tcgagttaGTCGAAACGACCTTGAGTTGTCGCATATTTg-3’
^46^KYd**A**tQgrfd^55^	T49A-S	5’-gatccaaatatgacGCGactcaaggtcgtttcgactaac-3’
	T49A-R	5’-tcgagttaGTCGAAACGACCTTGAGTCGCGTCATATTTg-3’
^46^KYdt**A**qGrfd^55^	T50A-S	5’-gatccaaatatgacacaGCGcaaggtcgtttcgactaac-3’
	T50A-R	5’-tcgagttaGTCGAAACGACCTTGCGCTGTGTCATATTTg-3’
^46^KYdtt**A**gRfd^55^	Q51A-S	5’-gatccaaatatgacacaactGCGggtcgtttcgactaac-3’
	Q51A-R	5’-tcgagttaGTCGAAACGACCCGCAGTTGTGTCATATTTg-3’
^46^KYdttq**A**rFd^55^	G52A-S	5’-gatccaaatatgacacaactcaagCGcgtttcgactaac-3’
	G52A-R	5’-tcgagttaGTCGAAACGCGCTTGAGTTGTGTCATATTTg-3’
^46^KYdttqg**A**fD^55^	R53A-S	5’-gatccaaatatgacacaactcaaggtGCGttcgactaac-3’
	R53A-R	5’-tcgagttaGTCGAACGCACCTTGAGTTGTGTCATATTTg-3’
^46^KYdttqgr**A**d^55^	F54A-S	5’-gatccaaatatgacacaactcaaggtcgtGCGgactaac-3’
	F54A-R	5’-tcgagttaGTCCGCACGACCTTGAGTTGTGTCATATTTg-3’
^46^KYdttqgrf**A** ^55^	D55A-S	5’-gatccaaatatgacacaactcaaggtcgtttcgCGtaac-3’
	D55A-R	5’-tcgagttaCGCGAAACGACCTTGAGTTGTGTCATATTTg-3’

Note: Introduced bases are shown in lowercase letters. The mutated amino acids are bold and underlined.

### SDS-PAGE and Western blot

Approximately equivalent amount of recombinant fusion protein was subjected to 12% sodium dodecyl sulfate-polyacrylamide gel electrophoresis (12% SDS-PAGE). The gel was either stained with commassie blue staining solution or electrophoretically transferred onto nitrocellulose membranes (BioRad, Mississauga, ON, Canada). PBS with 5% (w/v) skimmed milk was used to block the membranes. Following wash with PBST (0.5% Tween-20) three times, the membrane was incubated with primary antibody at a corresponding dilution in PBS for 1 h at room temperature. After wash three times with PBST, 100 μl HRP-conjugated goat anti-mouse IgG (Sigma, USA) was added at a corresponding dilution in PBS and incubated at 37°C for 1 h. The reaction was developed by 3, 3’-diaminobenzidine (DAB).

### Vaccine trial with the epitope peptide

To determine the protective immunity induced by the epitope peptide, each group including 10 BALB/c mice was immunized subcutaneously with 50 μg of GapC_1-150_ or GST-fusion epitope peptide emulsified with ISA50 V2, followed by two boosts with the same dose at 2-week intervals. Mice, immunized with the same amount of GST and PBS, were used as controls. Ten days after the final boost, all mice were intraperitoneally (i.p.) challenged with a lethal dose of *S*. *dysgalactiae* (5 × 10^7^ CFU/mouse). Animals were monitored on a daily basis and humanely euthanized when they exhibited defined humane endpoints.

### Statistical analysis

All data were analyzed by SAS software pack (SAS Institute, Cary, NC, USA). Comparisons between individual data points were made using a Student’s t-test, and *P* < 0.05 and *P* < 0.01 were regarded as statistically significance. Data were shown as means ± standard deviation.

## Results

### Expression and purification of recombinant protein

**Fig 1:**
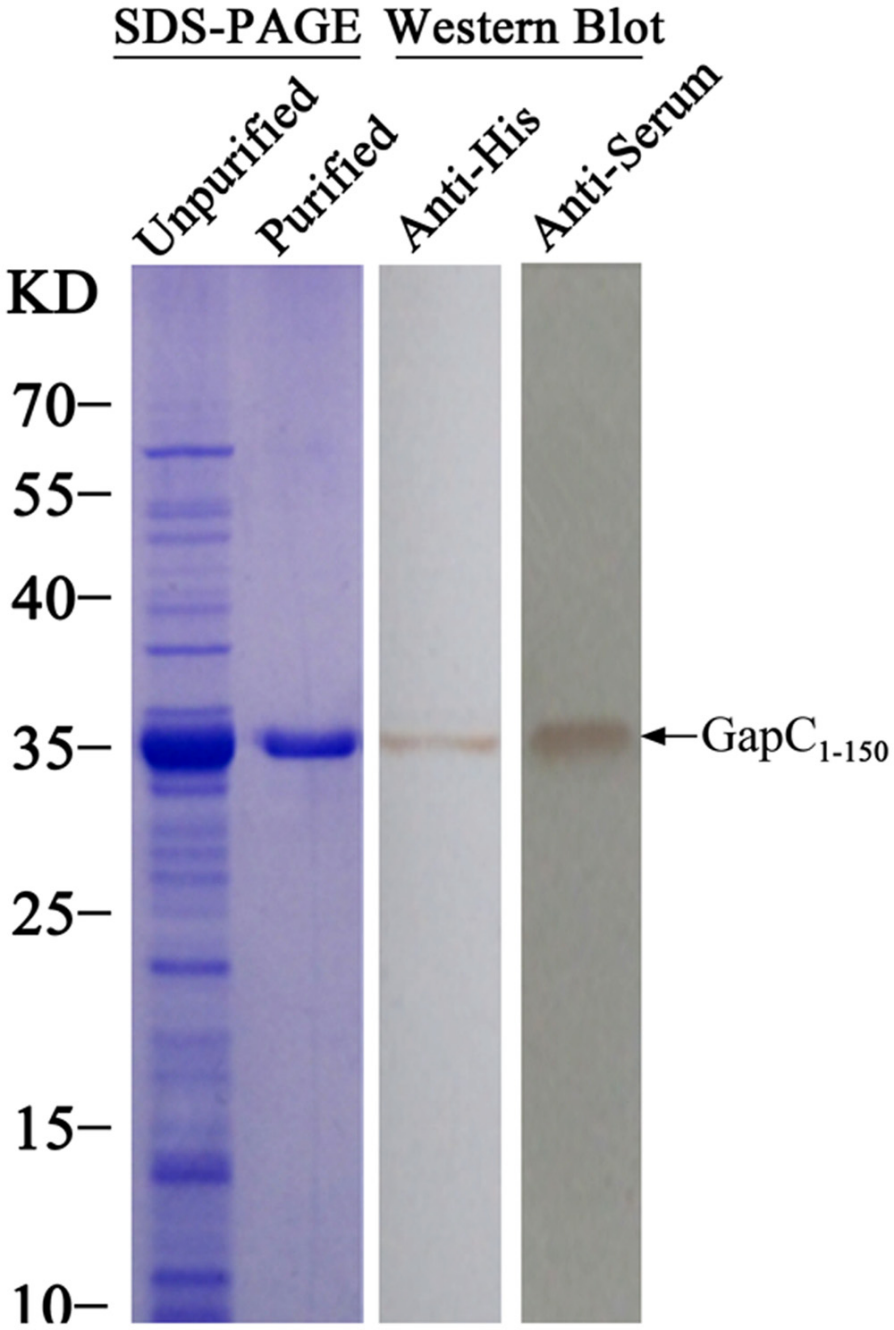
The recombinant GapC-His-TrxA protein was expressed and purified. The fusion protein was confirmed with Western Blot using anti-His antibody and anti-GapC serum, respectively.

The recombinant GapC_1-150_ was expressed as a His-TrxA fusion protein and purified by Ni-NTA purification system (Merck). The molecular weight of the recombinant GapC_1-150_ was approximate 35KD examined by 12% SDS-PAGE, in accordance with the molecular weight of the predicted protein. The recombinant GapC_1-150_ protein was further confirmed by Western blot using mouse anti-His monoclonal antibody and mouse GapC anti-serum, respectively (Fig 1). The results showed that the recombinant GapC_1-150_ was correctly expressed and well purified.

### Characterization of mAb5B7

**Fig 2:**
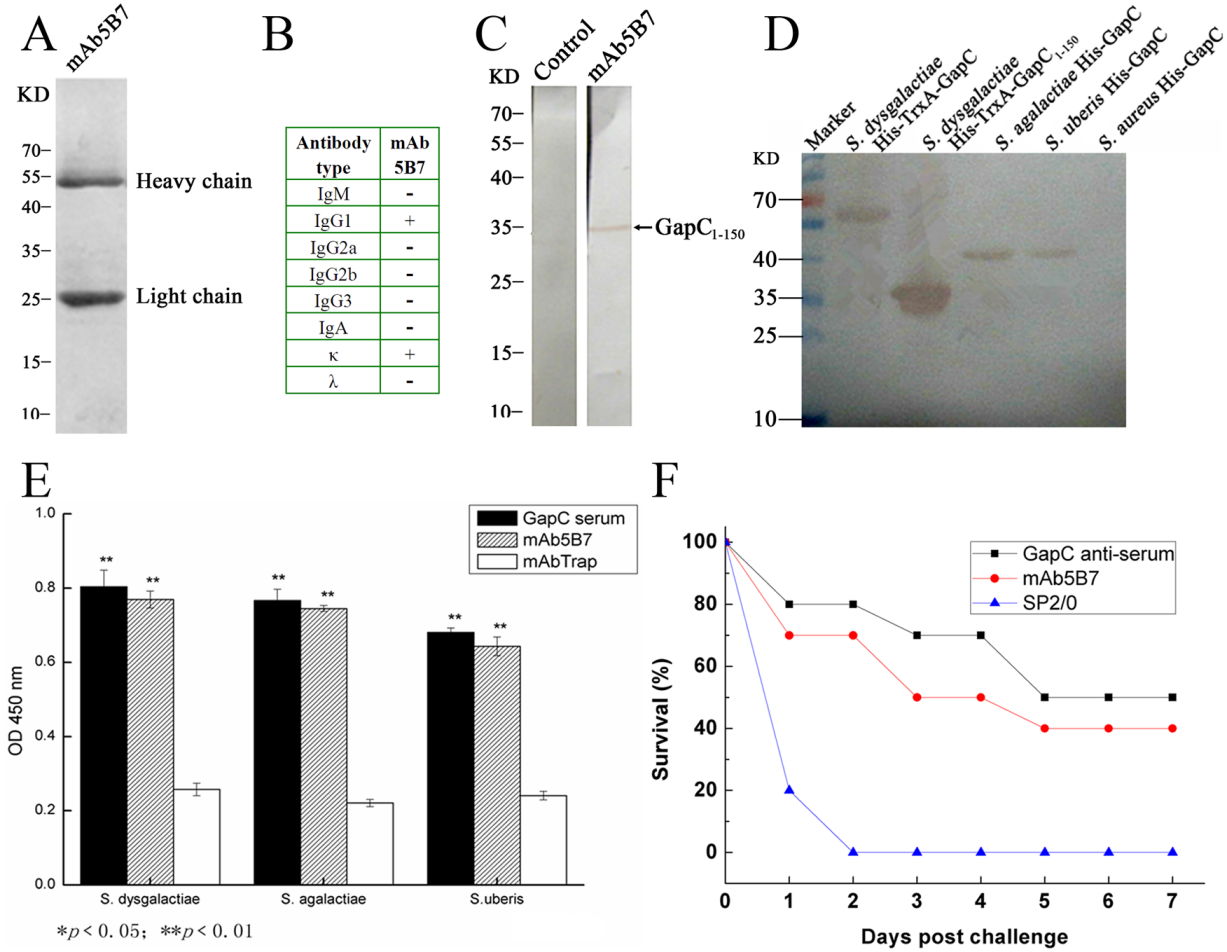
(A) The purified mAb5B7 was determined by SDS-PAGE. (B) The class of the mAb5B7 was determined to be IgG1 and κ chain using mouse mAb isotyping kit. (C) GapC^1-150^ recognized by mAb5B7 was detected using Western Blot. (D) The reactivity of mAb5B7 with the recombinant GapC of *S. dysgalactiae*, *S. uberis*, *S. agalactiae* and *S. aureus* was determined by Western blot. (E) The reactivity of mAb5B7 with the whole bacteria of inactivated *S. dysgalactiae*, *S. agalactiae* and *S. uberis* was confirmed by indirect ELISA (* *P* < 0.05; ** *P* < 0.01). (F) Passive immunization of mAb5B7 against *S. dysgalactiae* infection was performed and its protective effect was determined.

Three cell lines of hybridoma that secreted anti-GapC_1-150_ mAbs were established by the hybridoma technique. One of the cell lines, referred to as mAb5B7, could stably secrete anti-GapC antibody at high titer for more than 10 passages. Antibody titers of culture supernatant and ascites fluid were 1: 64000 and 1: 1.12 × 10^5^, respectively. The mAb5B7 against the recombinant GapC_1-150_ was successfully purified from mouse ascites (Fig 2A). The class of the mAb5B7 was determined to be IgG1 and κ chain (55KD, 26KD) (Fig 2B). The purified GapC_1-150_ could be probed with mAb5B7, but not with control normal ascites, confirming that GapC_1-150_ containing specific epitope recognized by mAb5B7 (Fig 2C).

The sequence homology of GapC_1-150_ protein among *S*. *dysgalactiae*, *S*. *agalactiae* and *S*. *uberis* is more than 93%. However, the *S*. *aureus* GapC_1-150_ displays a low degree of 63% sequence homology. Western blot analysis showed that mAb5B7 recognized recombinant GapC proteins of *S*. *dysgalactiae*, *S*. *agalactiae* and *S*. *uberis*, but did not recognize recombinant GapC protein of *S*. *aureus* (Fig 2D). The reactivity of mAb5B7 with the whole cells of the three *streptococcus* species was determined by indirect ELISA. The results showed that the mAb5B7 could significantly react with the whole cells of *S*. *dysgalactiae*, *S*. *agalactiae* and *S*. *uberis* (Fig 2E). These results implied that the epitope of three *streptococcus* species GapC_1-150_ was conserved and exposed on bacterial surface, which could be readily recognized by mAb5B7.

In order to investigate the protective effects of mAb5B7, passive immunization against *S*. *dysgalactiae* infection was performed. The results showed that all mice in control group were dead within 2 days. However, the mice immunized with mAb5B7 or GapC anti-serum survived for much longer time after *S*. *dysgalactiae* challenge (Fig 2F). These results implied that the mAb5B7 had a certain extent of immune protection against *S*. *dysgalactiae* infection, even not stronger than the GapC anti-serum.

### Epitopes prediction

**Fig 3:**
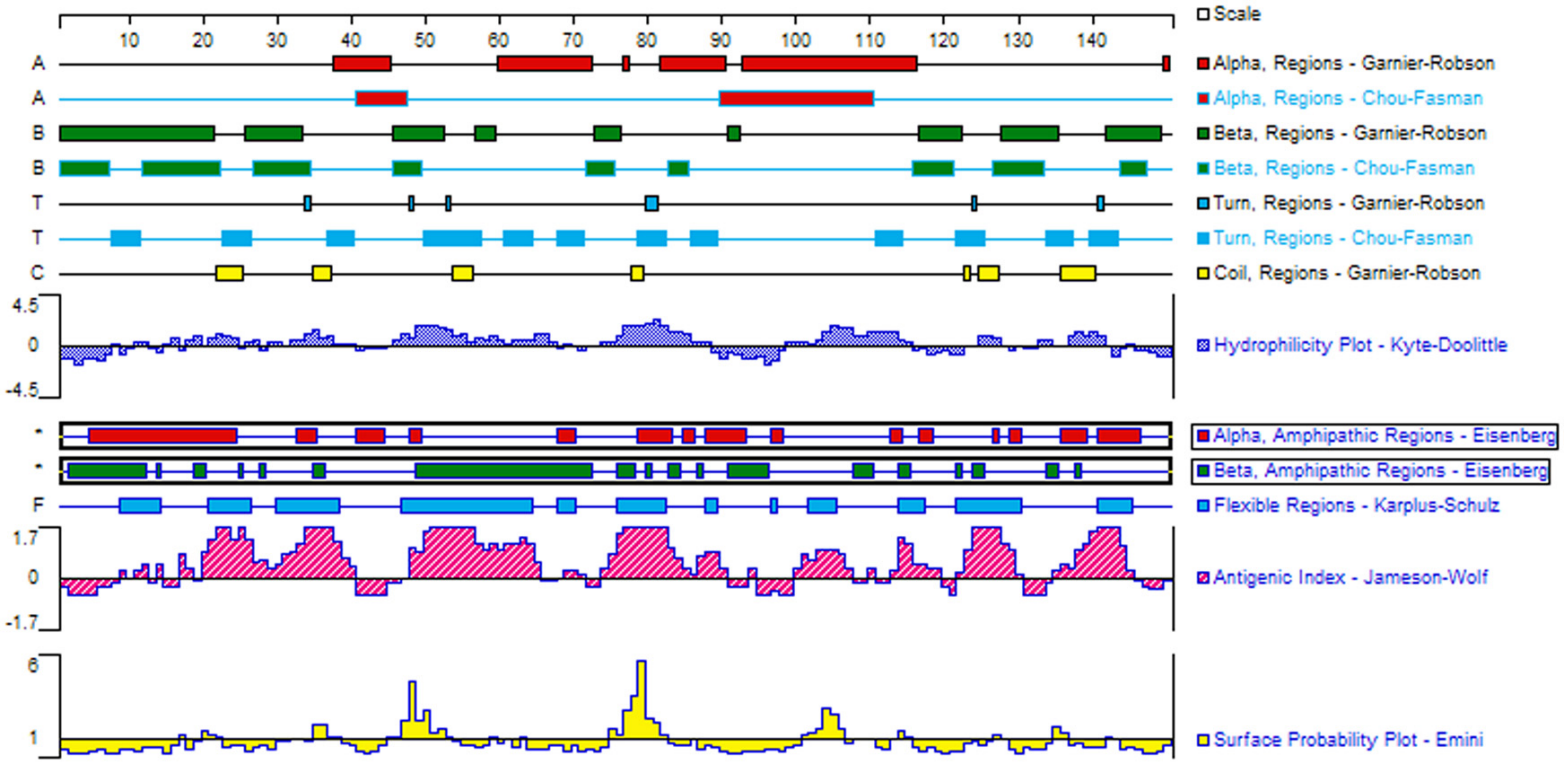
Secondary structures, flexibility, hydrophilicity, surface probability and antigenicity index for *S. dysgalactiae* GapC_1-50_ protein.

To further determine the epitopes of GapC and decrease the number of laboratory experiments, bioinformatics was used to predict the epitopes. The secondary structure and the surface properties of the GapC_1-150_ were analyzed (Fig 3). Based on these results, the most probable epitope regions recognized by mAb5B7 were within the N-terminus amino acids 35–37, 48–53, 78–82 or 102–107 of GapC_1-150_ protein.

### Phage-displayed epitope biopanned by mAb5B7

**Fig 4:**
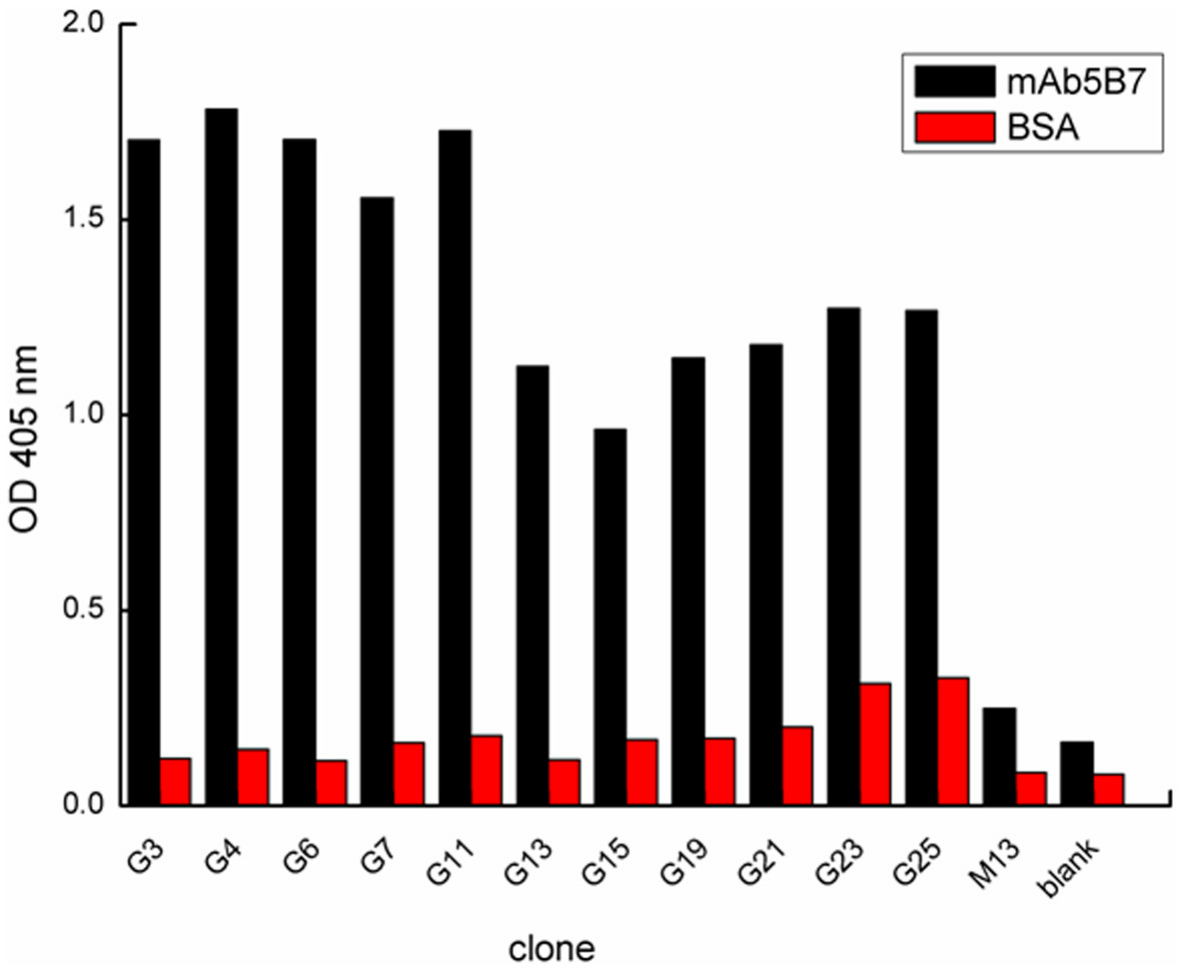
Detection of positive phage clones to mAb5B7 by sandwich ELISA. Wild-type M13 phage was used as a negative control. BSA-coated wells were used to exclude cross-activity. The *E. coli* ER2738 culture supernatant was blank control.

After three successive rounds of biopanning, the screened phages bound to the mAb5B7 were well enriched and yield of positive phage clones increased obviously (Table 2). Twenty-five clones selected by mAb5B7 were analyzed by sandwich ELISA, and eleven clones among them showed specific reactivity to mAb5B7 (Fig 4). The single-strand phage DNAs extracted from the 11 ELISA-confirmed positive clones was sequenced and the amino acid sequences were deduced. The amino acid sequences of the 11 clones showed a consensus motif DTTQGRFD (Table 3). The motif DTTQGRFD is located in amino acids 48–55 of the N-terminus of the *S*. *dysgalactiae* GapC_1-150_ protein. We propose that the motif°DTTQGRFD^55^ is an epitope of the *S*. *dysgalactiae* GapC_1-150_, which consistent with our prediction.

**Table 2:** Enrichment of positive phage clones by biopanning of Ph.D.-12 library.

Cycles	mAb (mg/L)	Washing (%TBST)	Input (pfu)	Output (pfu)	Yield
1	100	0.1	1.5 × 10^11^	7.1 × 10^5^	4.7 × 10^-6^
2	50	0.3	1.5 × 10^11^	8.1 × 10^8^	5.4 × 10^-3^
3	30	0.5	1.5 × 10^11^	1.0 × 10^9^	0.7 × 10^-3^

**Table 3:** Sequences of Ph.D.-12 phage-displaying peptides from the positive phage clones through bio-panning.

Phage	Amino acid sequence of the insert ^a^																
1					**D**	P	M	**Q**	N	D	**F**	T	M	G	A	N	
2	S	L	D	F	**D**	P	L	**Q**	**G**	**R**	W	**D**					
3			N	L	**D**	V	**T**	D	**G**	S	N	**D**	K	M			
4				A	A	P	Y	**Q**	**G**	L	M	G	A	H	A		
5				D	V	G	L	L	**G**	Q	**F**	A	G	Y	S		
6					S	H	**T**	**Q**	D	S	**F**	Q	M	D	Y	T	
7					S	H	**T**	**Q**	D	S	**F**	Q	M	D	Y	T	
8			S	C	H	K	**T**	E	**G**	W	C	T	R	H			
9	Q	Y	L	K	**D**	**T**	**T**	L	**G**	F	D	A					
10					T	**T**	L	E	**G**	**R**	Y	**D**	K	P	L	L	
11						N	M	L	**G**	M	Y	**D**	N	K	L	T	N
Consensus					**D**	**T**	**T**	**Q**	**G**	**R**	**F**	**D**					
GapC	L	L	K	Y	**D**	**T**	**T**	**Q**	**G**	**R**	**F**	**D**	G	T	V	E	V

^a^ Conservative amino acid motifs are bold and underlined.

### Precise defining of the epitope

**Fig 5:**
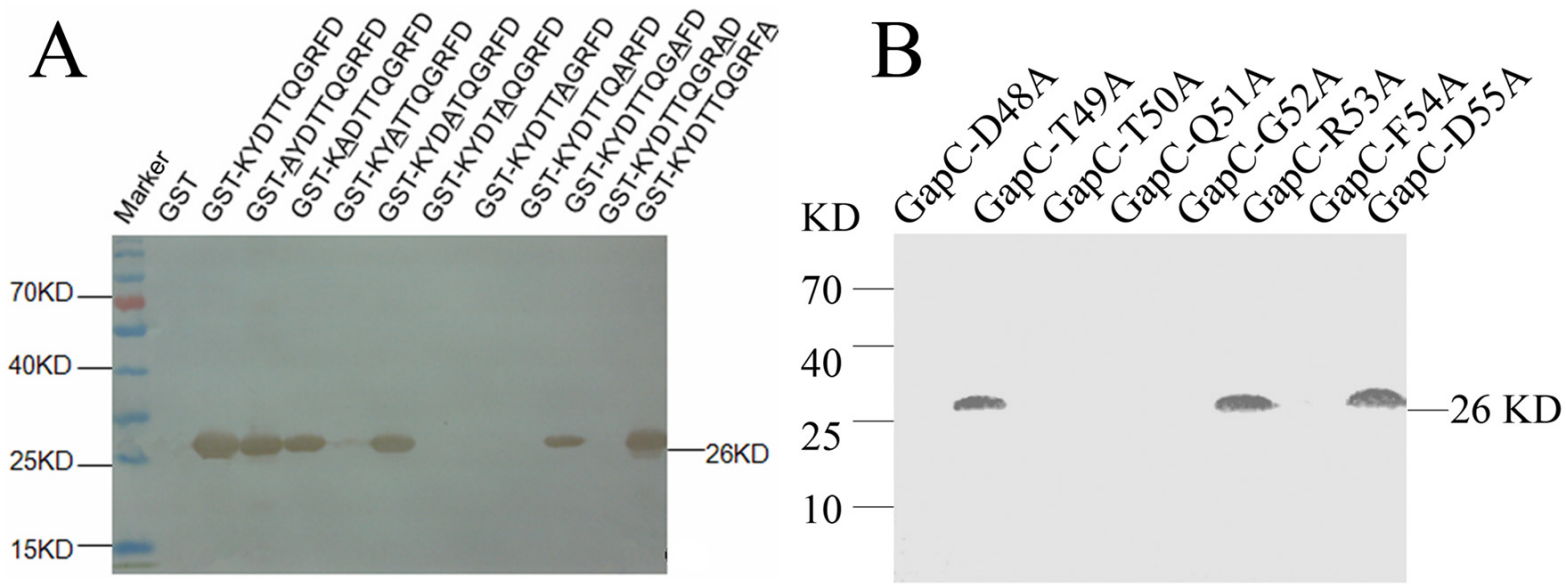
(A) The reactivity of various recombinant GST-fusion proteins with mAb5B7 was confirmed using Western blot. The mutant amino acids are underlined. (B) The reactivity of various mutant full-length GapC proteins with mAb5B7 was confirmed using Western blot. The mutant amino acids are highlighted with number.

To verify the epitope precisely, DNA fragments coding the motif ^48^DTTQGRFD^55^ and a series of mutated motifs AYDTTqgrfd, KADTTqgrfd, KYATTqgrfd, KYDATqgrfd, KYDTAqgrfd, KYDTTAgrfd, KYDTTqArfd, KYDTTqgAfd, KYDTTqgrAd, KYDTTqgrfA were expressed as GST-fusion protein, respectively. Western blot results showed that D48A, T50A, Q51A, G52A and F54A mutations completely destroyed the reactivity of the epitope with mAb5B7. However, the mutants R53A showed slightly reduced reactivity of the epitope with mAb5B7 (Fig 5A). In order to further determine the contribution of amino acids in the epitope peptide, we mutated each residue of the epitope to alanine in the full-length GapC, and determined their binding affinity with mAb5B7. The results showed that D48A, T50A, Q51A, G52A and F54A mutations abolished the reactivity of the epitope with mAb5B7 (Fig 5B). These results confirm that the motif ^48^DTTQGRFD^55^ is an authentic epitope in the GapC protein of *S*. *dysgalactiae* and the motif represents the minimal reactivity unit of the linear epitope recognized by mAb5B7.

### Alignment of the epitope sequence on GapC from different species

To analyze whether the linear epitope recognized by mAb5B7 is conserved, the epitope sequences of 7 *Streptococcus* species and 9 other microbial species are aligned. The results show that all amino acids in the motif are identical among all 7 *Streptococcus* species, suggesting that the motif ^48^DTTQGRFD^55^ represents a highly conserved epitope on the GapC protein of all *Streptococcus* species. However, the epitope motif of *Streptococcus* is different from the motif in other microbial species (Table 4).

**Table 4:** Alignment of the sequences surrounding the epitope-coding region on the GapC protein from different microbial species.

Species	EPITOPE MOTIF
*Streptococcus dysgalactiae* (AAP31408)	A H L L K Y **D T T Q G R F D** G T V E V
*Streptococcus agalactiae* (AAM73770)	A H L L K Y **D T T Q G R F D** G T V E V
*Streptococcus uberis* (AAM73771)	A H L L K Y **D T T Q G R F D** G T V E V
*Streptococcus iniae* (AAM73773)	A H L L K Y **D T T Q G R F D** G T V E V
*Streptococcus parauberis* (AAM73772)	A H L L K Y **D T T Q G R F D** G T V E V
*Streptococcus mutans* UA159 (AAN58118)	A H L L K Y **D S T Q G R F D** G N V E V
*Streptococcus sanguinis* SK340 (EGQ22894)	A H L L K Y **D T T Q G R F D** G T V E V
*Neisseria meningitides* alpha522 (CCA45832)	L H L F K Y **D S T Q G R F Q** G T A E L
*Staphylococcus epidermidis* M23864 (EFE58076)	A H L L K Y **D T** M **Q G R F T** G E V E V
*Staphylococcus aureus* (ABY73737)	A H L L K Y **D T** M **Q G R F T** G E V E V
*Zobellia galactanivorans* (YP_004735169)	L S H L L K Y **D S** I H **G** V **F** G A E V G
*Shigella sonnei* Ss046 (AAZ88415)	a y l l k h **D S** N Y **G** P F P W S V D F
*Clostridium acetobutylicum* EA (YP_005669854)	H L F K Y **D S S Q G R F** N **G** E I E V K
*Kingella kingae* ATCC 23330 (ZP_08468253)	V H L F K Y **D T T Q G R F D** G T V E L
Consensus	A H L L K Y **D T T Q G R F D** G T V E V

Note: The GenBank accession numbers of the used strains are indicated in parentheses. The homologous amino acid residues of the epitope motif are bold and underlined.

### Protective immunity elicited by epitope peptide

**Fig 6:**
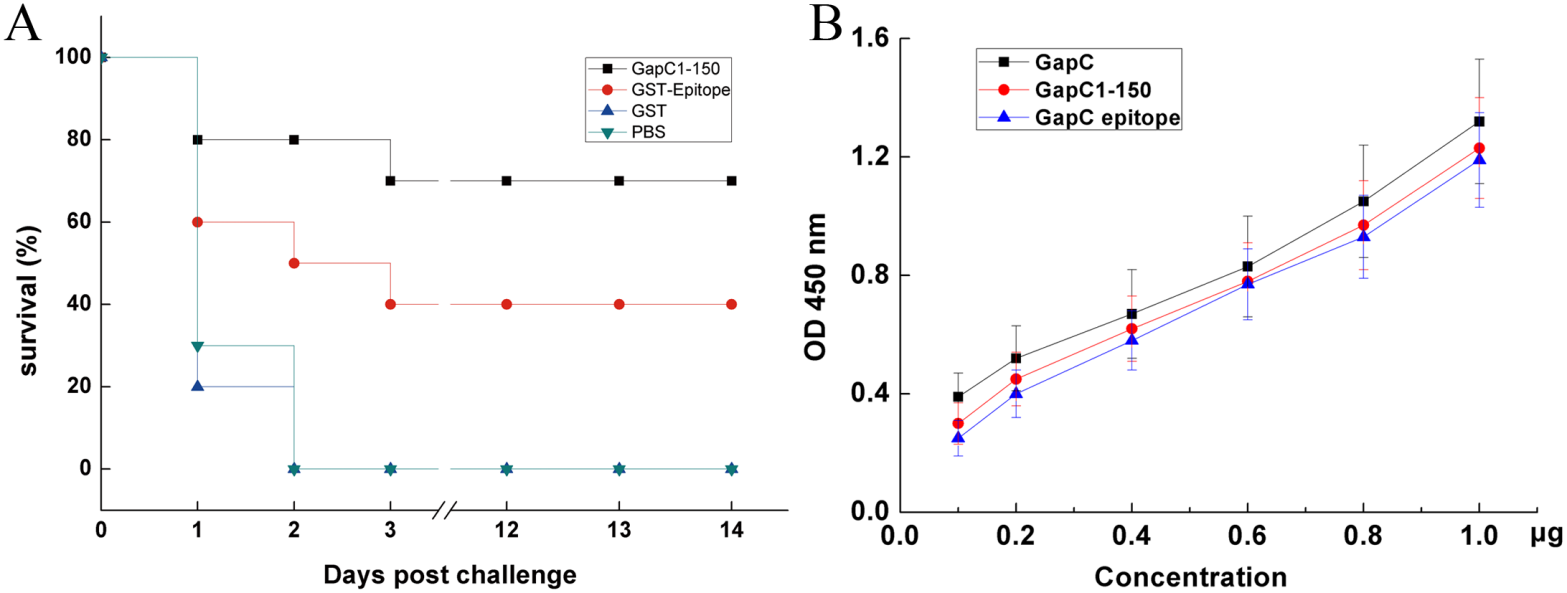
(A) Protective rate of immunized and control mice following challenge with *S*. *dysgalactiae* GapC. Immunized and control mice were challenged i.p. with *S*. *dysgalactiae* bacterial suspension (equivalent to 5 × 10^7^ CFU) on day 10 after the last immunization. Mice were monitored every day for day 60, and the data of day 14 were represented. (B) The difference in binding affinity of antibody mAb5B7 to the full-length GapC protein, GapC_1-150_ fragment and the GapC epitope fragment at concentration of 0.1–1.0 μg was determined with indirect ELISA.

To evaluate the immune responses of the epitope ^48^DTTQGRFD^55^, the purified GapC_1-150_ protein, GST-epitope peptide, GST and PBS-immunized mice were challenged (i.p) with *S*. *dysgalactiae* on day 10 after the last immunization. We first tested the antibody production in the immunized mice using indirect ELISA method, and the results indicated that antibody titer of the GapC_1-150_ protein and GST-epitope peptide was 1: 64000 and 1: 32000, respectively. Recombinant GapC_1-150_ had the lowest mortality of all groups, with a 70% survival rate. GST-epitope peptide-immunized mice had the second lowest mortality with 40% survival rate, although immune protection of epitope was not stronger than the GapC_1-150_. In contrast, GST protein and PBS couldn’t evoke protective immune responses, and no mice in two groups could survive via lethal challenge (Fig 6A). These results suggest that B-cell epitope elicits strong immune protection against *S*. *dysgalactiae* infection, and they also promote us to further investigate the difference in binding affinity of antibody mAb5B7 to the full-length GapC protein, GapC_1-150_ fragment and the GapC epitope fragment. The binding affinity was determined with indirect ELISA, and the results indicated that binding affinity of full-length GapC and GapC_1-150_ fragment was stronger than the GapC epitope fragment at concentration of 0.1–1.0 μg. Although the relative affinity on full-length GapC was slightly higher than GapC_1-150_ and GapC epitope fragments, there was not significant difference among these three different fragments (Fig 6B). These results are similar with the immune protection in animals dosed with the different fragments, suggesting that these fragments might evoker intensive immune response.

## Discussion

Elucidating the essential feature of a pathogen’s epitope recognized by antibodies can provide important information about the molecular mechanisms of immune [24]. Considerable evidence has been accumulated regarding the role of GapC protein in adhesion of *S*. *dysgalactiae* to host cells and pathogenesis, which is responsible for inducing protective immune response [13, 25]. Mapping the B-cell epitope of GapC is one of the crucial steps for the development of epitope-based vaccine against *S*. *dysgalactiae*. In this study, through screening phage-displayed random peptide 12-library with mAb5B7, we characterized a linear B-cell epitope in the GapC protein of *S*. *dysgalactiae*. The epitope matched to the consensus motif ^48^DTTQGRFD^55^, and site-directed mutagenic analysis confirmed that this core motif is the minimal requirement unit for the linearized epitope. Both mouse hyperimmune antisera and mAb5B7 against GapC showed strong reactivity with the identified B-cell epitope. More importantly, strong protective immunity was elicited by the epitope peptide.

Besides an important protein involving in glycolysis and pathogenesis, the GapC can also induce effective protection against *streptococcus* infection [8]. The functional and immunobiological characteristics of GapC make it a good candidate for the development of a prophylactic vaccine against *streptococcus* infection [5]. Through our previous experimental research, we confirmed that recombinant GapC could partially provide protection against *streptococcus* infection [18]. It is reported that monoclonal antibody technology are widely used to map B-cell epitopes [22, 26]. Subsequently, we seek to produce monoclonal antibody against GapC using hybridoma technique. One of cell clones, mAb5B7 with stable secretion of high titer antibody of IgG1 and κ, was obtained. The purified mAb5B7 could recognize the GapC proteins of *S*. *dysgalactiae*, *S*. *agalactiae* and *S*. *uberis*, but neither the GapC of *S*. *aureus* (Fig 2D) nor other recombinant proteins of *S*. *aureus* (data not shown), suggesting the reactivity between mAb5B7 and GapC was extremely specificity. The mAb5B7 can react with the whole cells of the three *streptococcus* species, indicating that the epitope recognized by the mAb5B7 is exposed on the bacterial surfaces. Furthermore, mAb5B7 possessed partial protection against *S*. *dysgalactiae* infection after passive transfer of the mAb5B7 into naive mice. These results showed that monoclonal antibody of GapC was successfully generated.

Bioinformatics method is an efficient way to predict epitope. Therefore, we used two online software programs (http://tools.immuneepitope.org/main/ and http://www.cbs.dtu.dk/services/BepiPred/) to predict the B-cell epitopes of the truncated GapC_1-150_ protein. The online prediction showed that the 30-50aa fragment of the GapC received the highest scores (Fig 3) and this fragment was determined as the continuous-epitope fragments. The phage displayed random peptide library is a vigorous tool for mapping B-cell epitopes recognized by monoclonal antibody [27]. In order to further verify the correctness of the above epitope prediction, we identified immunodominant B-cell epitopes within the truncated GapC_1-150_ by screening the Ph.D.-12 Phage Display Peptide Library using the mAb5B7. Through analysis of reactivity of the mAb5B7 to the positive phage clones and alignment of the positive phage sequences, a linear B-cell epitope recognized by mAb5B7 was defined as DTTQGRFD. This peptide sequence is identical to ^48^DTTQGRFD^55^, which is located in the N-terminus with GapC protein of *S*. *dysgalactiae* (Table 3). Furthermore, substitutions of the individual amino acids within the ^48^DTTQGRFD^55^ showed that residues D48A, T50A, Q51A, G52A and F54A mutations completely destroyed the reactivity with the mAb5B7. Thus, the peptide ^48^DTTQGRFD^55^ is determined to be the minimal unit of the epitope with the maximal binding activity to mAb5B7.

Lastly, sequences alignment of B-cell epitope was performed between *S*. *dysgalactiae* and other species in the genus *streptococcus*. The results indicate that *streptococcus* displays 100% sequence homology among various species, but the other bacteria possessing GapC protein display a low degree of sequence homology (Table 4). These results are consistent with the experimental data that the mAb5B7 react with the GapC proteins of *S*. *dysgalactiae*, *S*. *uberis*, *S*. *agalactiae*, but not react with GapC of *S*. *aureus*. Moreover, immunization with epitope peptide of GapC could significantly evoke protection against challenge of *S*. *dysgalactiae*. Therefore, it may be an effective antigenic epitope for further study of epitope-based vaccines against *streptococcus* infections.

## Conclusions

Taken together, we have identified a highly conserved linear B-cell epitope in the N-terminus of *S*. *dysgalactiae* GapC protein. These data may provide an explanation of the GapC-induced protection against three *streptococcus* species and highlight the possibility of developing the epitope-based vaccine against the *streptococcus*.
